# Increased aPKC Expression Correlates with Prostatic Adenocarcinoma Gleason Score and Tumor Stage in the Japanese Population

**DOI:** 10.1155/2014/481697

**Published:** 2014-04-29

**Authors:** Anthony S. Perry, Bungo Furusato, Raymond B. Nagle, Sourav Ghosh

**Affiliations:** ^1^Department of Pathology, Banner MD Anderson Cancer Center, Gilbert, AZ 85234, USA; ^2^Department of Pathology, Jikei University School of Medicine, Tokyo 105-8461, Japan; ^3^Department of Pathology, The University of Arizona and Arizona Cancer Center, Tucson, AZ 85724-5044, USA; ^4^Department of Cellular & Molecular Medicine, The University of Arizona and Arizona Cancer Center, Tucson, AZ 85724-5044, USA

## Abstract

*Background*. Levels of the protein kinase aPKC have been previously correlated with prostate cancer prognosis in a British cohort. However, prostate cancer incidence and progression rates, as well as genetic changes in this disease, show strong ethnic variance, particularly in Asian populations. *Objective*. The aim of this study was to validate association of aPKC expression with prostatic adenocarcinoma stages in a Japanese cohort. *Methods*. Tissue microarrays consisting of 142 malignant prostate cancer cases and 21 benign prostate tissues were subject to immunohistological staining for aPKC. aPKC staining intensity was scored by three independent pathologists and categorized as absent (0), dim (1+), intermediate (2+), and bright (3+). aPKC staining intensities were correlated with Gleason score and tumor stage. *Results*. Increased aPKC staining was observed in malignant prostate cancer, in comparison to benign tissue. Additionally, aPKC staining levels correlated with Gleason score and tumor stage. Our results extend the association of aPKC with prostate cancer to a Japanese population and establish the suitability of aPKC as a universal prostate cancer biomarker that performs consistently across ethnicities.

## 1. Introduction 

Unlike cancers that result from a specific susceptibility gene, such as chronic myeloid leukemia, prostate cancer correlates with changes in multiple loci, each with low individual risk but cumulatively leading to the prostate cancer phenotype. Ethnicity clearly affects prostate cancer risk. Prostate cancer is one of the most common malignancies in the United States and Western countries [1]. In contrast, the incidence of prostate cancer is significantly lower in Asian ethnicities, including Japanese [2, 3]. Prostate cancer incidence is approximately 2.8-fold higher in African-Americans than in Asians, while Caucasians constitute an intermediate risk group [4]. The mortality from prostate cancer is also significantly lower in the Japanese population when compared to Caucasians or African-Americans [2, 3]. However, Gleason scores at diagnosis are higher in Japanese men compared with Caucasians [3].

Prostate cancer can be frequently indolent and nonlethal; however it can be highly aggressive and lethal in some patients. The most appropriate treatment for patients with lower risk disease may be an active surveillance regimen in which they are spared from unnecessary procedures and treatments, improving their quality of life. At the same time, more lethal forms may require aggressive therapy including radical prostatectomy, external beam radiation therapy, and brachytherapy. The ability to accurately identify prostate cancer and discriminate lethal from nonlethal forms by pretreatment needle biopsy, through the use of biomarkers, may allow more informed treatment decisions in the management of the disease. Tissue biomarkers can act as an active surveillance tool discriminating insignificant disease from that with high risk of progression at the time of needle biopsy [5]. Such improved biomarker-guided prognosis and/or prediction of therapeutic response depend upon the identification of uniform and dependable molecular changes in the disease. Given the ethnic variation in prostate cancer progression, it is essential to know if a particular biomarker is of universal application with consistent performance characteristics across all ethnicities. Or perhaps the diagnostic and prognostic value of the biomarker would be restricted to a particular ethnicity.

The frequency of some genetic alterations in Asian versus Caucasian populations correlates with the incidence rate of prostate cancer, while others do not. For example, the frequency of 21q22.2-22.3 deletion (*TMPRSS2:ERG *fusion) and 10q deletion (*PTEN* inactivation) is significantly reduced in Chinese men, correlating with reduced prostate cancer incidence [6]. In contrast, allelic imbalance in 13q14 harboring tumor suppressors* RB1, DBM, FAM10A4, and FOXO1A *and 13q21 containing putative tumor suppressors* EDNRB *and* KLF5* was found to be higher in the Japanese population compared to a German cohort [3].

Th multifunctional serine-threonine protein kinase atypical protein kinase C (aPKC) consists of two ~70 kDa isoforms—aPKC zeta and iota (aPKC*ξ* and *ι*) and a ~55 kDa isoform named PKM*ξ* [7–9]. The two full-length isoforms, aPKC*ξ* and *ι*, have a high degree of overall sequence identity with ~86% amino acid identity in the enzymatic serine-threonine kinase domain. The truncated PKM*ξ* isoform is essentially identical to the aPKC*ξ* kinase domain. Expression and function of both the full-length isoforms have been associated with a number of cancers, including prostate cancer. Increased protein expression of aPKC*ξ* has been shown to strongly correlate with a subset of aggressive prostate cancers and inversely with patient survival [10, 11]. Expression of other, less characterized isoforms of aPKC has also been reported in prostate cancer [12]. Notwithstanding, aPKC*ξ* has also been described as a tumor suppressor in prostate cancer [13] and therefore the association of this kinase with prostate cancer remains controversial. Therefore, we sought to investigate if the reported increase in expression of aPKC isoforms in prostate cancer extends to a Japanese cohort or is subject to ethnic variations. 

## 2. Materials and Methods

### 2.1. Tissue Microarray (TMA) and Immunohistochemistry

All tissue collections were approved by Institutional Review Board and obtained with informed consent from patients. All tumor cases were histologically reviewed and representative tumor areas marked on the corresponding donor paraffin blocks. Tissue microarrays were prepared from archival blocks of formalin fixed paraffin embedded prostate specimens. Tissue of sufficient size (2 mm) was cored from representative areas for TMA. Clinical and pathologic data were obtained from medical records. 3 *μ*m sections were cut for routine hematoxylin and eosin (H&E) staining and immunohistochemistry (IHC). IHC on the TMAs was done at TACMASS Core (Tissue Acquisition and Cellular/Molecular Analysis Shared Service), Arizona Cancer Center. IHC was performed with rabbit anti-aPKC (sc-216) primary antibody purchased from Santa Cruz Biotechnology, Inc. (Dallas, Texas), on a Discovery XT Automated Immunostainer (VMSI—Ventana Medical Systems, a Member of the Roche Group, Tucson, Arizona). This antibody recognizes all identified aPKC isoforms [14]. All steps were performed on Discovery XT Automated Immunostainer using VMSI validated reagents, including deparaffinization, cell conditioning (antigen retrieval with a borate-EDTA buffer), primary antibody staining, detection and amplification using a biotinylated-streptavidin-HRP and DAB (Diaminobenzidine) system, and hematoxylin counterstaining. Following staining on the instrument, slides were dehydrated through graded alcohols to xylene and coverslipped with mounting medium. Primary antibody was used at a 1 : 150 dilution and incubation time was 1 h at 37°C. Appropriate positive and negative (secondary antibody only) controls were stained in parallel for each round of immunohistochemistry.

### 2.2. Statistical Analyses

Statistical significance was determined by appropriate tests using SPSS and Prism. Distribution of samples within each aPKC staining category was analyzed by Chi-squared test.

## 3. Results

We used tissue microarrays (TMA) prepared from specimens from Japanese patients undergoing prostatectomy following Institutional Review Board approval. A total of 163 specimens were included after assessment by participating pathologists for Gleason and TNM (tumor, lymph node, and metastasis) score. 142 cases identified as malignant prostate cancer were included in this study and 8 of those were diagnosed as metastatic prostate cancer. Additionally, 21 benign samples were included as controls. The age distribution of the patient population and the number of specimens of different Gleason scores and TNM staging are described in Table 1. The TMAs were stained as described in Section 2 and examined by three pathologists independently for the level of aPKC protein expression. aPKC expression levels were binned in four categories—absent (0), dim (1+), intermediate (2+), and bright (3+). In case of differential scoring of an individual specimen, at least two pathologists reviewed and came to consensus to determine the final score.

**Figure 1:**
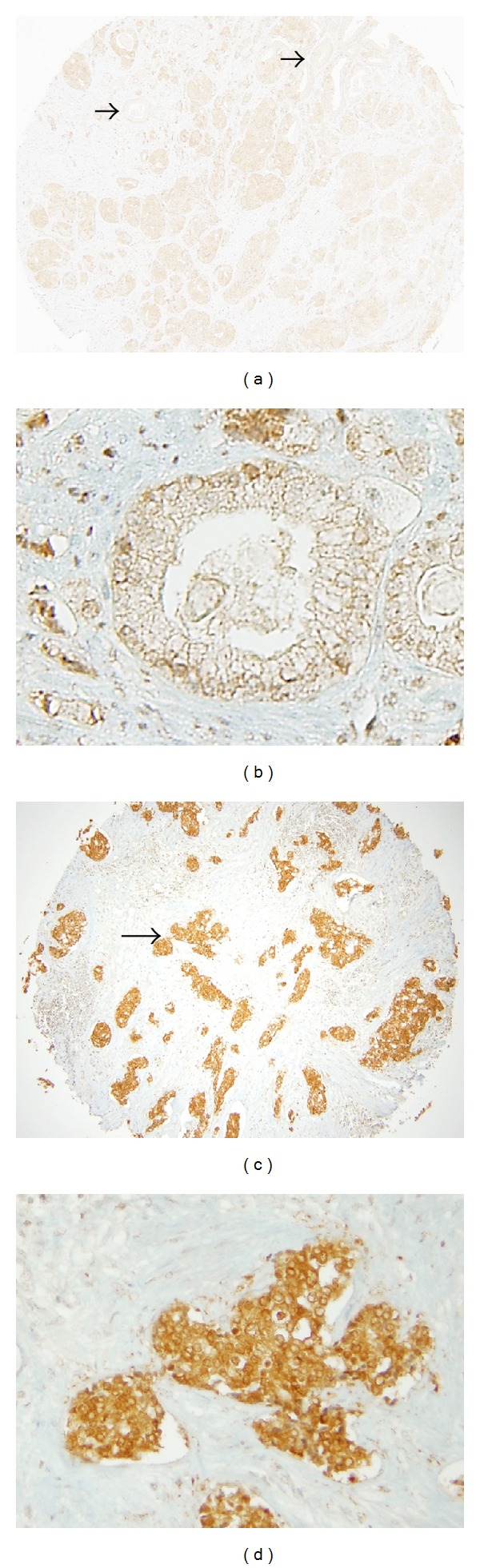
aPKC staining in prostatic tissue. (a and c) Representative images of aPKC staining in TMA cores of prostatic tissue. Arrows in (a) point to normal glands, and in (c) to malignant region (b) Magnified view of a normal gland showing dim (1+) aPKC staining. (d) Magnified view of malignant region showing bright (3+) aPKC staining.

**Figure 2:**
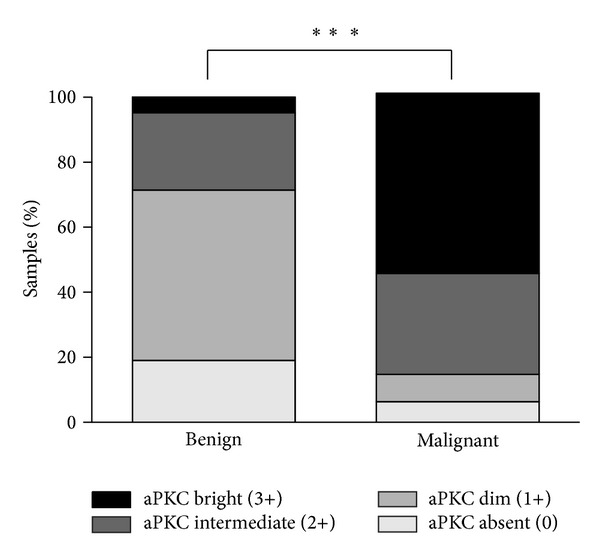
Association of aPKC staining intensity with malignant prostate cancer. Distribution of aPKC staining intensity (absent or 0, dim or 1+, intermediate or 2+, and bright or 3+) for benign (*n* = 21) and malignant (*n* = 142) prostate cancers. ***P < 0.001 Chi-square test.

Examination of aPKC protein level in prostate cancer demonstrated that benign control tissue, as well as benign glands found within prostate cancer tissue, did not show significant aPKC staining and were described either as aPKC absent (0) or aPKC dim (1+) (Figure 1(a), shown with arrow; Figure 1(b)). Scoring the TMA revealed that 19.06% of benign samples were aPKC absent (0) and 52.38% were aPKC dim (1+). 23.81% of benign cases were aPKC intermediate (2+) and 4.76% were aPKC bright (3+) (Figure 2). In contrast, malignant prostate cancer tissue showed increased aPKC staining (Figures 1(c) and 1(d)). Only 6.34% of cases scored as aPKC absent (0) and 8.45% scored aPKC dim (1+). 30.99% of cases were aPKC intermediate (2+) and 54.23% of cases were aPKC bright (3+) in malignant prostate cancer (Figure 2). Of note, the few adenocarcinomas with absent (0) or dim (1+) aPKC were also morphologically undifferentiated. The distribution of samples, in both benign tissue and malignant prostate cancer, within different aPKC staining categories was not random (Chi-square test, *P* < 0.001). Comparison of the distribution of samples in different aPKC staining categories between benign tissue and malignant prostate cancer revealed a significant positive correlation (Chi squared test, *P* < 0.001). Therefore, we conclude that aPKC expression is significantly higher in prostate cancer from the Japanese population.

**Figure 3:**
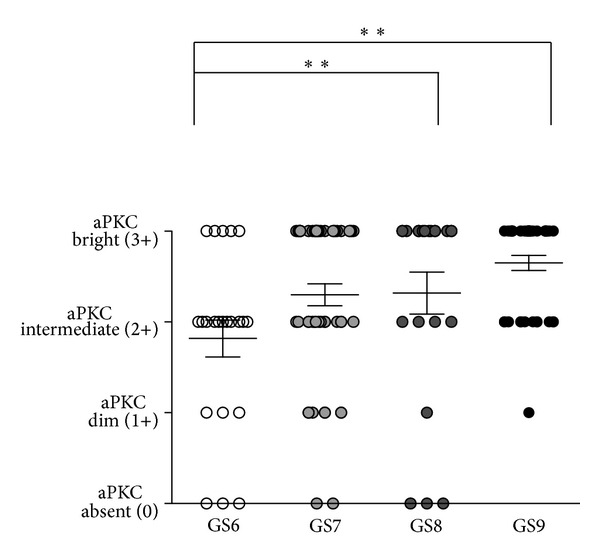
Correlation of aPKC staining intensity with Gleason scores. Distribution of aPKC staining intensity in prostate cancers of different Gleason scores (GS). **P < 0.01, Chi-square test. Overall, aPKC scores correlated with GS, Chi-square test, *P* < 0.05.

The Gleason score is the most commonly used prostate cancer staging system and presently the best prognostic criteria in prostate cancer [15]. Previously, aPKC expression has been shown to correlate individually with Gleason score and the prognosis of prostate cancer in a British cohort of undetermined ethnicity [10, 16, 17]. Therefore, we compared the Gleason score of the TMA specimens with the aPKC scores. Our analysis demonstrates that aPKC scores correlate with Gleason score (Chi-square test) (Figure 3). Furthermore, Gleason 6 most frequently had an intermediate (2+) aPKC expression level. Furthermore, Gleason 7–9 scores correlated with higher aPKC expression levels, with aPKC protein levels being highest in Gleason score 9 (Figure 3). The aPKC score in Gleason scores 8 and 9 prostate cancer was significantly different than in prostate cancer tissue of Gleason score 6 (Chi-square test). Overall, lower Gleason scores tended to be aPKC dim (1+), but, as scores lower than 6 are uncommonly diagnosed, we did not have sufficient numbers to evaluate significance.

**Figure 4:**
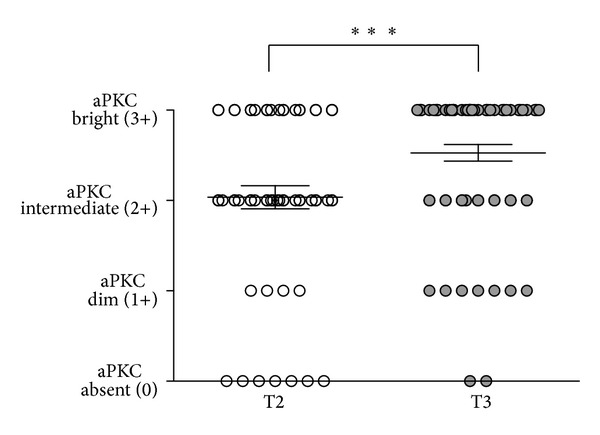
Correlation of aPKC staining intensity with tumor stage. Distribution of aPKC staining intensity in prostate cancer at different tumor stages (T). ***P < 0.001, Chi-square test. Overall, aPKC score correlated with T, Chi-square test, *P* < 0.01.

We also analyzed if aPKC expression levels correlate with tumor stage (T), lymph node metastasis (N) or distant metastasis (M). aPKC scores correlated with T stages (Chi-square test). We observed that T2 cases were predominantly aPKC intermediate (2+), while T3 cases were aPKC bright (3+) (Figure 4). The aPKC score in T2 was significantly lower than in T3 (Chi-square test). T4 cases also showed bright (3+) aPKC expression levels, but our sample size was limiting for statistical significance. In contrast, no definitive correlation can be established with N or M score of the prostate cancer specimens, as higher scores are uncommonly diagnosed, and we did not have sufficient numbers to evaluate significance. In conclusion, we observed a correlation of aPKC expression level with tumor stage.

## 4. Discussion

The prevalence and prognosis of prostate cancer varies with ethnicity. Lifestyle and environmental factors may account for ethnic differences in this disease; however key genetic differences have also been reported [3]. Prostate cancer in the Japanese population shows interesting genetic differences from the Caucasian population [3]. Therefore, we tested whether increased aPKC protein levels, previously described in prostate cancer, are observed in Japanese patients with prostate cancer. Our results indicate that aPKC expression was significantly increased in prostate cancer versus normal prostate tissue in Japanese patients. These results support the previous report of increased aPKC phosphorylation at the PDK1 site, which correlates with increased activity, in hormone-naïve and castration resistant prostate cancers in a Japanese cohort [18]. Furthermore, we extend these observations to an association of increased aPKC expression levels with Gleason score and tumor stage.

Our studies are also consistent with the reports ascribing a direct molecular function of aPKC*ξ* in tumor progression. Androgens stimulate aPKC*ξ* activity in the androgen-dependent phase of this disease, while aPKC*ξ* is constitutively activated in androgen-insensitive prostate cancer [19]. aPKC*ξ* stimulates proliferation by activating p70S6 kinase. Furthermore, inhibition of aPKC kinase activity results in reduced proliferation of both androgen-dependent and androgen-insensitive prostate cancer cells [19]. In prostate cancer cell lines, aPKC*ξ* is activated by Src-Rac1 signaling [18]. Additionally, conserved molecular function of aPKC*ξ* in cell migration [18] and aPKC*ι* in NF-*κ*B activation and increased cell survival have been reported in prostate cancer cells [20]. Taken together, our results indicate that aPKC may be a universal biomarker suitable for prostate cancer detection and staging, unaffected by ethnic genetic differences observed in prostate cancer.

## Figures and Tables

**Table 1:** Patient age and tumor characteristics of the TMA.

Age	
Mean	65.92
Median	67
Minimum	20
Maximum	87
Gleason score	
6	22
7	50
8	22
9	40
10	8
Stage	
T2	57
T3	74
T4	11
N0	123
N1	19
M0	136
M1	6
